# Comparative efficacy of the Cognitive Behavioral Analysis System of Psychotherapy versus Supportive Psychotherapy for early onset chronic depression: design and rationale of a multisite randomized controlled trial

**DOI:** 10.1186/1471-244X-11-134

**Published:** 2011-08-17

**Authors:** Elisabeth Schramm, Martin Hautzinger, Ingo Zobel, Levente Kriston, Mathias Berger, Martin Härter

**Affiliations:** 1 Department of Psychiatry and Psychotherapy, University Medical Center Freiburg, Hauptstraße 5, 79104 Freiburg, Germany; 2 Department of Psychology, University of Tuebingen, Christophstr. 2, 72072 Tuebingen, Germany; 3 Department of Medical Psychology, University Medical Center Hamburg-Eppendorf, Martinistraße 52, 20246 Hamburg, Germany

## Abstract

**Background:**

Effective treatment strategies for chronic depression are urgently needed since it is not only a common and particularly disabling disorder, but is also considered treatment resistant by most clinicians. There are only a few studies on chronic depression indicating that traditional psycho- and pharmacological interventions are not as effective as in acute, episodic depression. Current medications are no more effective than those introduced 50 years ago whereas the only psychotherapy developed specifically for the subgroup of chronic depression, the Cognitive Behavioral Analysis System of Psychotherapy (CBASP), faired well in one large trial. However, CBASP has never been directly compared to a non-specific control treatment.

**Methods/Design:**

The present article describes the study protocol of a multisite parallel-group randomized controlled trial in Germany. The purpose of the study is to estimate the efficacy of CBASP compared to supportive psychotherapy in 268 non-medicated early-onset chronically depressed outpatients. The intervention includes 20 weeks of acute treatment with 24 individual sessions followed by 28 weeks of continuation treatment with another 8 sessions. Depressive symptoms are evaluated 20 weeks after randomisation by means of the 24-item Hamilton Rating Scale of Depression (HRSD). Secondary endpoints are depressive symptoms after 12 and 48 weeks, and remission after 12, 20, and 48 weeks. Primary outcome will be analysed using analysis of covariance (ANCOVA) controlled for pre-treatment scores and site. Analyses of continuous secondary variables will be performed using linear mixed models. For remission rates, chi-squared tests and logistic regression will be applied.

**Discussion:**

The study evaluates the comparative effects of a disorder-specific psychotherapy and a well designed non-specific psychological approach in the acute and continuation treatment phase in a large sample of early-onset chronically depressed patients.

**Trial registration:**

ClinicalTrials.gov (NCT00970437).

## Background

Chronic depressions account for roughly up to a third of all mood disorders [1,2]. In the literature, four subtypes of chronic depression are distinguished: (1) dysthymia, (2) chronic major depression, (3) recurrent major depression with incomplete remission during episodes, and (4) double depression [3]. Dysthymic disorder is defined as a mild condition that persists for at least 2 years. Major depressive episode, chronic type, refers to a more severe condition that meets full criteria for major depression for a minimum of 2 subsequent years. Patients who no longer meet full criteria for a major depressive episode but continue to experience significant symptoms for a total duration greater than 2 years are referred to as recurrent major depression with incomplete remission during episodes. The superimposition of a major depressive episode on antecedent dysthymia is termed as double depression [3]. The lifetime prevalence rate for dysthymia in the US is estimated to be 6% and the one-year prevalence rate around 3% [4]. The mean length of the chronic course is approximately 17 to 30 years [5,2]. It is a particularly disabling disorder which is associated with greater comorbidity, more significant impairments in functioning, increased health care utilization, and more frequent suicide attempts and hospitalizations than acute major depressive episodes [1]. It therefore accounts for a considerable proportion of the enormous economic burden associated with depression [6]. In more than 70% of all cases, chronic depression begins early in life [7], is mostly associated with early interpersonal trauma and often persists life long. *Early-onset chronic depression *results in an even more substantial human capital loss compared with late-onset [8]. In addition, the disorder has a more malignant course than the late-onset group [9] and shows a high rate of relapse after an initial response to medication treatment [10].

Although psychotherapy is commonly applied to chronically depressed patients with early-onset there is little data on the efficacy of psychological interventions in this population. In a recent meta-analysis, Cuijpers et al. [11] concluded that psychotherapy is effective in the treatment of chronic depression and dysthymia but probably less than pharmacotherapy. However, most of the studies had methodological weaknesses such as the very short courses of psychotherapy. Indications were found that at least 18 treatment sessions are needed to realize optimal effects of psychotherapy [11].

One large study (n = 681) [12] showed in a re-analysis [13] that for the subgroup of chronically depressed patients with an early childhood trauma, a psychotherapeutic method specifically designed for chronic depression (Cognitive Behavioral Analysis System of Psychotherapy/CBASP) was particularly effective. In contrast, medication (nefazodone) alone had a weak effect in this subgroup of patients as only 33% reached remission (48% with CBASP). The combination of CBASP and nefazodone resulted in a higher remission rate (54%) than both monotherapies. Similarly, imipramine but also more traditional psychotherapies (Interpersonal Psychotherapy/IPT, and Cognitive Behavioral Therapy) performed relatively poorly in early-onset chronic Major Depression as shown by a reanalysis [14] of the data of the NIMH-Collaborative study. In a pilot study [15] with 30 chronically depressed outpatients with early onset, statistically significant differences were found between CBASP and IPT regarding remission rates (57% in CBASP vs. 20% in IPT) and the reduction of self-rated depressive symptoms in favor of CBASP. The benefits of CBASP over IPT might have been due to the extended course of therapy (22 sessions) and sample characteristics (82% early traumatized subjects).

Based on these results, CBASP with and without medication seems to be a highly promising method for early onset chronic depression. Yet, CBASP was never compared as a first-line treatment to a non-specific psychological intervention (active control). A control treatment consisting of active but non-specific supportive psychotherapy (SP) was used by Markowitz et al. [16] with early-onset dysthymic patients as a comparator to IPT. Supportive psychotherapy did perform as well as IPT (both with low remission rates of 12% and 22%) supporting the argument of Wampold et al. [17] that psychological "placebos" - when properly designed and structurally equivalent - are as effective as psychotherapy in depressive disorders. This argument is supported by a recent trial [18] where CBASP did not prove superior to SP when applied as a short-term (12 sessions) augmentation strategy in chronically depressed patients showing partial or non-response to a medication algorithm. In summary, the available data support the need for more and larger trials, controlling for medication, and including CBASP as a powerful intervention with a more intensive (larger number of sessions) and a longer course of treatment to unfold beneficial and lasting effects in chronic depression.

## Objectives

The purpose of the study is to estimate the efficacy of CBASP compared to non-specific supportive psychotherapy (SP) in 268 medication free, early onset chronically depressed outpatients. The primary hypothesis is that CBASP is more effective in reducing depressive symptoms than SP. The secondary hypothesis assumes that the remission rates are higher in CBASP compared to SP.

## Methods/Design

### Study design

**Figure 1:**
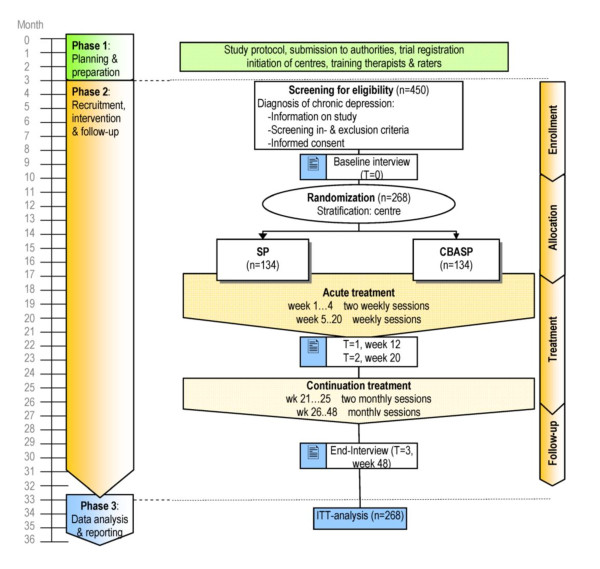
**Trial design**. CBASP: Cognitive Behavioral Analysis System of Psychotherapy. SP: Supportive Psychotherapy. Wk: week.

This investigator-initiated trial is registered as: "A comparison of the Cognitive Behavioural Analysis System of Psychotherapy against supportive psychotherapy for early onset chronic depression" at ClinicalTrials.gov (NCT00970437). Figure 1 illustrates the study design of this multisite, observer blind, prospective, parallel-group, randomized, controlled trial with an active control and two treatment phases (acute and continuation). A stratified block randomization with randomly varying block size is performed, stratified by trial center.

The intervention includes 20 weeks of acute treatment with 24 individual sessions followed by 28 weeks of continuation treatment with another 8 sessions in each arm of the trial.

### Oversight of research with human participants

The study is being conducted in compliance with the protocol, Good Clinical Practice and the applicable regulatory requirements. This research was approved by the Institutional Review Board/Institutional Ethical Committee (IRB/IEC) of the University of Freiburg and the ongoing trial is under continued review by the IRB/IEC. The local IRB/IECs of each participating site were asked for confirmation prior to initiation. Written informed consent is provided by all participants prior to clinical interview, randomization and intervention.

Adverse Events and Serious Adverse Event (AE/SAE) are reported to the project's Data Safety and Monitoring Board (DSMB) and SAEs to each IRB/IEC. Criteria for discontinuation include for individuals: a) active suicidality; b) the physical health of the patient is at risk according to clinical judgment; c) occurrence of an AE/SAE with therapeutic implications incompatible with the study; d) newly occurring exclusion criteria, or e) the informed consent is withdrawn. Parts of the trial or the entire trial will be discontinued if: a) an investigator has serious ethical concerns because of the performance at one of the sites. b) severe safety concerns become apparent to the DSMB.

In addition, the DSMB will conduct regular phone conferences and visits at trial sites to ensure compliance with ethical principles and the study protocol, as well as to check data quality and accuracy.

### Data collection sites

The patients are recruited and treated at eight clinical sites in Germany:

1) Department of Clinical and Developmental Psychology, University of Tuebingen, (Site Principal Investigator: Martin Hautzinger, PhD);

2) Department of Psychiatry, University of Heidelberg (Site Principal Investigator: Matthias Backenstraß, PhD);

3) Central Institute of Mental Health in Mannheim (Site Principal Investigator: Josef Bailer, PhD);

4) Psychological Outpatient Clinic, University of Marburg, (Site Principal Investigator: Katrin Wambach, PhD);

5) Department of Psychiatry and Psychotherapy, University of Luebeck, Germany (Site Principal Investigator: Philipp Klein, MD);

6) Department of Psychiatry; University Medical Center Bonn (Site Principal Investigator: Dieter Schoepf, MD);

7) Department of Psychosomatic Medicine and Psychotherapy, University Medical Center Hamburg-Eppendorf and Clinic Center Eilbek (Site Principal Investigator: Bernd Löwe, MD)

8) Department of Psychiatry and Psychotherapy, University Medical Center Freiburg (Site Principal Investigator: Elisabeth Schramm, PhD).

Recruitment methods include project promotion through reports in the media and by letters/announcement to private practitioners. Most patients are expected to be referred from practitioners. Patients have to be unmedicated for at least 2 weeks prior to study entry. In addition, the treating or a collaborating physician has to attest that there is no contraindication for psychotherapeutic treatment. When a review of medical history necessitates doing so, a physical examination and appropriate laboratory tests are obtained to ensure that patients are diagnostically eligible. Patient recruitment started in March 2010.

All Site Principal Investigators will continue to meet regularly by telephone (at least monthly or if necessary more frequently) and at least twice annually in person until study completion. Each research procedure is described in detail in a procedure manual to facilitate the implementation of the procedures in a consistent manner across sites.

### Inclusion and exclusion criteria

Key inclusion criteria are:

- a DSM-IV diagnosis of: 1) chronic Major Depressive Disorder/MDD (at least 2 years duration), or 2) current MDD superimposed on a pre-existing dysthymic disorder (so called "double-depression"), or 3) recurrent MDD with incomplete remission between episodes. Further inclusion criteria are:

- early onset (before the age of 21) according to DSM-IV,

- age between 18 and 65, and

- a score of at least 20 on the 24-item Hamilton Rating Scale of Depression (HRSD).

Key exclusion criteria include:

- acute risk for suicide (as opposed to suicidal thoughts) assessed according to clinical practice guidelines; suicidal patients are eligible, as long as outpatient treatment is deemed safe by the clinician,

- a history of psychotic symptoms, bipolar disorder, or organic brain disorders,

- a primary diagnosis of another axis I disorder,

- antisocial, schizotypical, or borderline personality disorder (SCID-II),

- severe cognitive impairment,

- a serious medical condition,

- absence of a response to a previous adequate trial of CBASP and/or SP, and

- other ongoing psychotherapy or medication.

### Interventions

CBASP as the experimental intervention follows a manual [19,20] and is a highly structured approach based on an interpersonal contemporary learning acquisition model [21]. It is the only psychotherapy model developed exclusively for the treatment of chronic forms of early-onset depression. The founder of the approach, James McCullough, suggests that chronically depressed patients suffer from a reduction of perceived functionality, i.e. the ability to detect the effects of their own behavior on other persons. This results in a pervasive degree of social isolation, which worsens the depressive mood. In addition, in chronic depressives early interpersonal traumatization resulted in an inhibition of maturation in childhood. CBASP focuses on the patient's central mechanisms of derailed affective and motivational regulation by using the therapeutic relationship in a personal, disciplined way to shape dysfunctional interpersonal behaviour. Further specific techniques (e.g. Interpersonal Discrimination Exercise, Situation Analysis) are applied to aid acquisition of perceived functionality in the patient. In summary, CBASP integrates behavioral, cognitive, and interpersonal strategies to help the patient recognizing the consequences of their behavior and interacting more effectively with others. Other goals of CBASP include the transferal of social problem solving strategies to the daily living, the interpersonal healing of earlier trauma and to generate authentic empathy.

The alternative treatment, supportive psychotherapy, is an active but less specific, manualized [22] control intervention previously used in several comparative trials [23]. SP - defined as non-interpersonal and non-cognitive-behavioral therapy - resembles supportive clinical management or client-centered counseling, and includes psychoeducational elements. SP is also defined as a psychotherapy wherein the therapist strives to create a supportive relationship by emphasizing non-specific therapeutic interactions and techniques that convey to the patient the therapist's interest, concern, and understanding. It utilizes the so-called common factors that have been assumed to account for much of the effect of all tested psychotherapies. These common or non-specific factors include the facilitation of affect, helping the patient to feel understood, to provide a framework for understanding, empathy, a treatment ritual, success experiences, hope and therapeutic optimism. It is assumed that many clinicians in private practice proceed in this unstructured manner. The explanatory mechanism for treatment effect offered to the patient focuses on the antidepressive effects of a supportive and understanding relationship, on the benefits of exploring and expressing emotions, the patient-directed structure of session and focus on personally relevant themes.

The number and duration of sessions as well as the experience of the therapists is equivalent in both study conditions. According to a meta-analysis of Baskin et al. [24] structurally equivalent "placebos" produced negligible effects compared to active treatments [25]. But there are also studies with depressed subjects which show significant effects for supportive interventions [26,17]. Both CBASP and SP are conducted with two weekly individual sessions of 50 minutes each for the first 4 weeks and 1 weekly session for the remaining 16 weeks in the acute phase (=24 sessions, see Figure 1), followed by 8 continuation sessions over the next 28 weeks (2 sessions in the first 4 weeks, and 1 session every 4 weeks thereafter). A 12-months naturalistic follow-up is planned for a second study phase since sustainment of response is particularly relevant given the chronic nature of the disorder.

Medication/treatments during trial: In cases of severe sleep problems, zolpidem is allowed for a maximum of 3 weeks. Central acting drugs are not allowed during the study. Single dosages of non-steroid analgesics and other non-centrally acting drugs for medical conditions are permitted.

### Description of risks

Psychotherapeutic treatment with CBASP as well as with SP involves the chance of improvement of the depressive symptomatology. Side effects of evidence-based psychotherapies are fortunately rather rare [27]. Possible undesired „side-effects" may include transient worsening of symptoms and transient risk of suicidality at the beginning of therapy (due to breaking out of avoidance behavior), but was rarely observed [28].

### Therapist training and monitoring of adherence

CBASP and SP are implemented by two separate groups of psychotherapists, both trained (in a 2-day training workshop and at least 1 practice day) in one of the methods and meeting the criteria for mastery of CBASP or SP procedures as assessed by evaluation of their performance during two videotaped pilot cases. All psychotherapists have completed a 3-year psychotherapy training program or are in an advanced stage of training. All sessions will be videotaped and site-supervisors continue to review the videotapes regularly on a random basis to assess psychotherapists' adherence to the treatment procedures using specific rating scales [19,29]. In addition, a separate team of independent raters trained to reliability will randomly evaluate several of the tapes from early, middle, and late therapy phase of each treatment for adherence and therapist competence. Site-supervisors will be directly supervised by the trial-supervisors (E. Schramm & M. Hautzinger) in terms of bi-weekly conference calls and meetings (twice a year or more if needed).

### Outcome measures

#### Severity of depression

The 24-item-version of the HRSD has been implemented in the study by Keller et al. [12] and will therefore provide internationally comparable study results. In addition, the applied 16-item Quick Inventory of Depressive Symptomatology, clinician-rated (QIDS-C16) and the self-rated 30-item Inventory of Depressive Symptomatology, Self-Report (IDS-SR) [30] have highly acceptable psychometric properties [31].

#### Treatment expectation

In a modified version of the IDS-SR (E-IDS), patients will be asked what they expect to answer in the IDS-SR at the end of therapy.

#### Severity of anxiety

In order to identify anxiety as a possible predictor for treatment outcome, symptoms of anxiety will be measured using the anxiety scale and the phobic anxiety scale from the Brief Symptom Inventory (BSI). The BSI is a validated self-report scale with strong test-retest and internal consistency reliabilities. Factor analytic studies of the internal structure of the scale have demonstrated its construct validity [32]. Further, the GAD-7 [33] is used. It is a valid and efficient tool with 7 items for screening for generalized anxiety disorder.

#### Quality of life

The Medical Outcome Study 36-item Short Form Health Survey (SF-36) [34] is an internationally approved, generic instrument to assess Health-Related Quality of Life (HRQoL). The 12-item Short Form Survey (SF-12), derived from the SF-36, has been demonstrated to be reliable and valid in clinical and population-based applications in the U.S. and other countries [35-38]. A more disease-specific instrument is the Quality of Life in Depression Scale (QLDS). This 34-item measure was developed to measure the impact of depression symptoms and treatment on quality of life [39]. The QLDS has evidence of reliability, construct and content validity, and sensitivity to change in depressed patients [40].

#### Interpersonal problems

Interpersonal problems will be measured with the German translation of the 64-item self-report Inventory of Interpersonal Problems (IIP-64) [41]. Psychometric research on the instrument in English-speaking communities as well as in German-speaking populations [41] demonstrated the validity and the reliability (good internal consistency and test-retest reliability) of the IIP-64.

#### Social functioning

To measure global psychological, social, and occupational functioning, the widely utilized Global Assessment of Functioning (GAF, Axis V in DSM-IV) scale will be used. Another, more specific measurement is the Social Adaptation Self-Evaluation Scale (SASS) [42], particularly for self-assessment of social functioning by patients with depression. It contains 21 items covering the different aspects of social interactions, global social attitude, and self-perception. The SASS has been validated and found to be simple to use and sensitive to changes in the different areas of social functioning [43].

#### Childhood trauma

At baseline, the Childhood Trauma Questionnaire (CTQ) [44] will be completed. The CTQ is a 28-item retrospective self-report questionnaire which determines 4 severity categories of emotional/physical/sexual abuse, and emotional/physical neglect. "Early trauma" is defined as one of these experiences before the age of 18 to a degree of at least "moderate to severe". In addition, the Early Trauma Inventory (ETI) [45], a 56-item semi-structured interview is used. The ETI assesses also the domain of general trauma (not assessed by CTQ). The psychometric properties of both instruments have shown to be favorable [46].

#### Evaluation by a relative

At the end of the therapy, a relative of the patient will be asked to evaluate 14 items of depressive symptoms before and after therapy.

#### Process analyses of therapies

After each session, the patients and therapists will fill out the Helping Alliance questionnaire (HAQ) developed by Luborsky [47]. The instrument assesses two types of alliance: patient's experience of feeling helped and supported by the therapist and patient's experience of working together with the therapist in a joint effort in order to overcome the difficulties. The HAQ is correlated with other well validated instruments [48], is also highly correlated with treatment outcome [49] and shows similar psychometric properties to other alliance instruments [50].

#### Screening, socio-demographic, and medical data

The initial screening visit consists of a medical and psychiatric history. Diagnoses will be derived using the Structured Clinical Interview for DSM-IV (SCID-I and II) [51,52] during the screening evaluation as well as after 20 and 48 weeks of treatment. Sociodemographic data include sex, age, nationality, marital status, education, occupation, measure of household income, and employment. Medical data refer to previous or present diseases, outpatient and/or inpatient psychiatric and/or psychotherapeutic treatments; suicidal attempts and risk factors for suicide.

### Endpoints

#### Primary endpoint

Depressive symptoms 20 weeks after randomization (after acute treatment phase) as measured by the 24-item HRSD.

#### Secondary endpoints

Among others:

a) Depressive symptoms after 12 and 48 weeks measured by the HRSD;

b) Remission rates after 12, 20, and 48 weeks utilizing the IDS-SR and defining remission a priori as a score of 13 or less for at least three consecutive weeks.

c) HRSD-remission rates (HRSD ≤ 8) and HRSD-response rates (HRSD score by at least 50 percent from baseline) will be calculated for the main measurement time points.

d) Changes in *QIDS-C16 *(from baseline to week 12, 20, and 48);

e) Temporal changes in *IDS-SR *- Total sum score between baseline assessment and follow-up assessments (time course of 27 time points).

### Measures taken to minimize/avoid bias

#### Randomization

The internet-based randomization will be conducted according to a central computerized randomization schedule, with a 1:1 treatment allocation ratio, stratified by centre, in blocks of variable size, to guarantee concealment. No-one can delete records from the randomization database, so that all randomizations have to be accounted for. Audit log files detailing all activity on the randomization system are available to the trial coordinator.

#### Blinding

All clinical ratings will be completed by trained and independent evaluators blinded to treatment assignment. Each of the sites implements procedures to mask a patient treatment assignment from the person who will evaluate the results of the clinical ratings through the following: 1) locating the rater at a separate physical location, and 2) reminding the patients at each visit not to mention anything that might reveal their treatment condition to the independent evaluator. The baseline and the HRSD interview at 20 weeks are videotaped and will be evaluated by another rater.

#### Control of therapy allegiance

Several recommendations for how to minimize the allegiance effect are considered: involving several investigators who represent a "mix of therapy allegiances", comparing interventions of the same length and duration, using blinded raters for process and outcome analyses, and conducting both interventions in all sites [53].

#### Control for overlapping treatments

The following measures are taken to prevent confounding of treatment conditions through the overlap of treatment methods:

Each therapist will conduct only one of the two treatments. The therapists are obligated to adhere to the therapeutic procedures and interventions described within the manuals. Adherence to the treatment manuals will be continuously supervised by rating videotapes of the sessions on a randomized basis using adherence scales [19,29]. The validity of the therapist's statements will be checked through external assessment of the video recordings. In the post-hoc-analysis it will be checked if the expected differences regarding intervention characteristics appear within the therapies.

#### Control for confounding factors

The influence of the trial site upon the effectiveness of the respective treatment approaches will be investigated as a separate factor. In addition, patients are asked not to engage in off-study psychosocial (e.g., group therapy) or psychiatric interventions (e.g. antidepressive medication) during the treatment period.

### Statistical methods

#### Power calculation

The sample size calculation is based on the primary hypothesis testing CPASP against SP with regard to mean HRSD-scores at the end of the acute treatment phase (null hypothesis: identical expected HRSD). We considered a difference of five points on the HRSD between mean post-treatment scores after 24 sessions of the treated groups as clinically relevant. In similar studies, standard deviations of post-intervention HRSD-scores of groups receiving CBASP, SP, or combinations range from 5.4 to 10.4 points [12,16]. Assuming a common standard deviation of 10 points for two-group comparisons yields a medium-sized effect of 0.5 (Cohen's d). To detect this effect by a two-tailed t-test with a power (1-β) of 0.95 and type I error probability level of α = 0.05 for significance, 210 patients (105 per group) are needed.

Assuming a drop-out rate of approximately 20% from baseline to week 20, the maximum sample size is fixed as 268 patients to be randomized. This is the number needed for an appropriately powered per-protocol analysis (only completers are analyzed). In the intention-to-treat analysis, the higher number of patients is expected to be compensated by a potential dilution of treatment effects, so that the power will be approximately the same.

#### Analyses

The final analyses will be performed in the intention to treat (ITT) population, analyzing patients in treatment groups to which they were randomized, and using the last observation carried forward (LOCF) method in case of missing outcome data at week 20. Because of the chronic nature of the disorder, spontaneous remission is unlikely to happen.

At main analysis, the null hypothesis of equal efficacy will be tested (two-sided test) using analysis of covariance (ANCOVA) controlled for pre-treatment scores and site. Secondary analyses of the primary endpoint will include a per-protocol approach, regression controlling for additional factors, and exploratory analyses of treatment effect modifiers. To examine changes over time, a mixed model approach to repeated measures with 2 treatments by 4 measurement points will be used: baseline, after 12 weeks, after 20 weeks (acute intervention), and after 28 further weeks of continuation treatment. Analyses of continuous secondary variables will be performed using linear mixed models. For remission rates, chi-squared tests and logistic regression will be used. The analysis of time to remission and time to response will be analyzed using standard survival analysis techniques. Level of significance will be set at α = .05

## Discussion

Specific and effective treatment strategies for chronic depression are urgently needed since the disorder is not only recognized as highly prevalent and particularly impairing, but is also considered "difficult-to-treat" or even treatment resistant by most clinicians. There are only a few studies on chronic depression indicating that traditional interventions are less effective than in acute, episodic depression. In addition, most of the studies had methodological weaknesses, such as the very short courses of psychotherapy. Usually, chronic depression begins early in life, is often associated with early interpersonal trauma, and the early onset course results in an even more substantial human capital loss than the late-onset. Furthermore, early onset depression shows a weak response to medication and a high rate of relapse after an initial response. Innovative antidepressant approaches should aim at the regulation and maintenance of mood stability over the long term rather than at the acute resolution of symptoms. The only specific and promising psychological intervention for chronic depression, the CBASP, focuses on the patient's central mechanisms of derailed affective and motivational regulation by using the therapeutic relationship in a personal, disciplined way to shape dysfunctional interpersonal behaviour. With the present multisite study, the efficacy of CBASP is compared with a non-specific supportive psychotherapy (a well-designed psychological control treatment) in early onset chronically depressed patients. The CBASP approach faired very well in one large trial but has never been directly compared to a non-specific control as first-line treatment. Another innovative aspect of the study is the use of an extended course of psychotherapy (32 sessions) since the very short courses of psychotherapy in the study by Keller et al. [12] and in other trials on chronic depression were probably too short to provide an adequate test of psychotherapy. Furthermore, it is planned to identify predictors of response to CBASP vs. SP (e.g. early childhood trauma). In process research, we will investigate which elements of psychotherapy are most critical for an antidepressant response to further improve the approach, if indicated.

### Strengths and limitations

Even though the combination of psycho- and pharmacotherapy is the recommended gold standard for the treatment of chronic depression in guidelines [54], to test the relative efficacy of this very condition would require a pharmacological placebo arm which would be ethically questionable. The same is true for medication alone. We consider medication alone also a relevant treatment option since it is probably the most frequently used strategy in clinical practice and maybe superior to psychological interventions [11]. However, evidence shows that it had a weak effect in the subgroup of early onset chronic depression [13]. Furthermore, this type of disorder shows a high rate of relapse after an initial response to medication alone [7]. Given the long duration of the trial it would be unethical to keep non-responsive patients on medication alone for 20 weeks. In addition, the focus of the present trial is on the proof of the CBASP-concept and its utility as a psychological method and not on the comparable efficacy of CBASP vs. medication. For the same reason and since it does not reflect clinical practice, we do not use a CBASP plus placebo condition. Thus, instead of trying to answer all questions with one design we focus on a specific aim addressing the efficacy of CBASP vs. supportive psychotherapy.

In this trial the requirements for a low risk of bias (sometimes also termed as "high methodological quality") are met. Randomization, blinding of raters, control of therapy allegiance and of overlapping treatments as well as for confounding factors are described and warranted by corresponding measures. The sample size is large and the different orientation of the sites (dept. of psychology, psychosomatic medicine, and psychiatry and psychotherapy) allows the generalization of the results.

## Competing interests

The authors declare that they have no competing interests.

## Authors' contributions

ES formulated the research question, the conception and design of the study, and drafted the manuscript. MHA participated in the development of the research design and revised the manuscript substantially. IZ reviewed existing literature, made significant contributions to formulating the research question, participated in the design and coordination of the study, and helped to draft the manuscript. LK defined the statistical methods. MB provided administrative support and has been involved in drafting the manuscript. MHÄ made substantial contributions to the conception and research design, and provided a critical revision of the manuscript for important intellectual content. All authors read and approved the final version of the manuscript.

## Pre-publication history

The pre-publication history for this paper can be accessed here:

http://www.biomedcentral.com/1471-244X/11/134/prepub

## References

[B1] Arnow BA Constantino MJ Effectiveness of psychotherapy and combination treatment for chronic depression J Clin Psychol 2003 59 8 893 905 10.1002/jclp.10181 12858430

[B2] Gilmer WS Trivedi MH Rush AJ Wisniewski SR Luther J Howland RH Yohanna D Khan A Alpert J Factors associated with chronic depressive episodes: a preliminary report from the STAR-D project Acta Psychiatr Scand 2005 112 425 433 10.1111/j.1600-0447.2005.00633.x 16279871

[B3] Klein DN Chronic Depression: Diagnosis and Classification Curr Dir Psychol Sci 2010 19 96 100 10.1177/0963721410366007

[B4] Kessler RC McGonagle KA Zhao S Nelson CB Hughes M Eshleman S Wittchen HU Kendler KS Lifetime and 12-month prevalence of DSM-III-R psychiatric disorders in the United States. Results from the National Comorbidity Survey Arch Gen Psychiatry 1994 51 1 8 19 8279933 10.1001/archpsyc.1994.03950010008002

[B5] Kocsis JH Gelenberg AJ Rothbaum B Klein DN Trivedi MH Manber R Keller MB Howland R Thase ME Chronic forms of major depression are still undertreated in the 21st century: Systematic assessment of 801 patients presenting for treatment J Affect Disord 2008 110 55 61 10.1016/j.jad.2008.01.002 18272232 PMC3515672

[B6] Greenberg PE Kessler RC Birnbaum HG Leong SA Lowe SW Berglund PA Corey-Lisle PK The economic burden of depression in the United States: how did it change between 1990 and 2000? J Clin Psychiatry 2003 64 12 1465 1475 10.4088/JCP.v64n1211 14728109

[B7] Cassano GB Akiskal HS Perugi G Musetti L Savino M The importance of measures of affective temperaments in genetic studies of mood disorders J Psychiatr Res 1992 26 4 257 268 10.1016/0022-3956(92)90032-J 1491352

[B8] Berndt ER Koran LM Finkelstein SN Gelenberg AJ Kornstein SG Miller IM Thase ME Trapp GA Keller MB Lost human capital from early-onset chronic depression Am J Psychiatry 2000 157 6 940 947 10.1176/appi.ajp.157.6.940 10831474

[B9] Klein DN Schatzberg AF McCullough JP Dowling F Goodman D Howland RH Markowitz JC Smith C Thase ME Rush AJ LaVange L Harrison WM Keller MB Age of onset in chronic major depression: relation to demographic and clinical variables, family history, and treatment response J Affect Disord 1999 55 2-3 149 157 10.1016/S0165-0327(99)00020-8 10628884

[B10] Agosti V One year clinical and psychosocial outcomes of early-onset chronic depression J Affect Disord 1999 54 1-2 171 175 10.1016/S0165-0327(98)00040-8 10403160

[B11] Cuijpers P van Straten A Schuurmans J van Oppen P Hollon SD Andersson G Psychotherapy for chronic major depression and dysthymia: A meta-analysis Clin Psychol Rev 2010 30 1 51 62 10.1016/j.cpr.2009.09.003 19781837

[B12] Keller MB McCullough JP Klein DN Arnow B Dunner DL Gelenberg AJ Markowitz JC Nemeroff CB Russell JM Thase ME Trivedi MH Zajecka J A comparison of nefazodone, the cognitive behavioral-analysis system of psychotherapy, and their combination for the treatment of chronic depression N Engl J Med 2000 342 20 1462 1470 10.1056/NEJM200005183422001 10816183

[B13] Nemeroff CB Heim CM Thase ME Klein DN Rush AJ Schatzberg AF Ninan PT McCullough JP Jr Weiss PM Dunner DL Rothbaum BO Kornstein S Keitner G Keller MB Differential responses to psychotherapy versus pharmacotherapy in patients with chronic forms of major depression and childhood trauma Proc Natl Acad Sci USA 2003 100 24 14293 14296 10.1073/pnas.2336126100 14615578 PMC283585

[B14] Agosti V Ocepek-Welikson K The efficacy of imipramine and psychotherapy in early-onset chronic depression: a reanalysis of the National Institute of Mental health Treatment of Depression Collaborative Research Program J Affect Disord 1997 43 3 181 186 10.1016/S0165-0327(97)01428-6 9186788

[B15] Schramm E Zobel I Dykierek P Kech S Brakemeier EL Külz A Berger M Cognitive behavioral analysis system of psychotherapy versus interpersonal psychotherapy for early-onset chronic depression: A randomized pilot study J Affect Disord 2011 129 1-3 109 116 10.1016/j.jad.2010.08.003 20822814

[B16] Markowitz JC Kocsis JH Bleiberg KL Christos PJ Sacks M A comparative trial of psychotherapy and pharmacotherapy for "pure" dysthymic patients J Affect Disord 2005 89 1-3 167 175 10.1016/j.jad.2005.10.001 16263177

[B17] Wampold BE Minami T Tierney SC Baskin TW Bhati KS The placebo is powerful: estimating placebo effects in medicine and psychotherapy from randomized clinical trials J Clin Psychol 2005 61 7 835 854 10.1002/jclp.20129 15827993

[B18] Kocsis JH Gelenberg AJ Rothbaum BO Klein DN Trivedi MH Manber R Keller MB Leon AC Wisniewski SR Arnow BA Markowitz JC Thase ME Cognitive Behavioral Analysis System of Psychotherapy and Brief Supportive Psychotherapy for Augmentation of Antidepressant Nonresponse in Chronic Depression Arch Gen Psych 2009 66 11 1178 1188 10.1001/archgenpsychiatry.2009.144 PMC3512199 19884606

[B19] McCullough JP Treatment for Chronic Depression. Cognitive Behavioral Analysis System of Psychotherapy 2000 New York: Guilford Press 10.1002/jclp.10176 12858425

[B20] Schramm E Schweiger U Hohagen F Berger M Psychotherapie für chronische Depression. Cognitive Behavioral Analysis System of Psychotherapy (CBASP) von James P. McCullough 2006 München: Elsevier21425638

[B21] McCullough JP CBASP, the Third Wave and the treatment of chronic depression Journal of European Psychotherapy 2010 9 169 190

[B22] Markowitz JC Manber R Rosen P Therapists' responses to training in brief supportive psychotherapy Am J Psychother 2008 62 1 67 81 18461844 10.1176/appi.psychotherapy.2008.62.1.67

[B23] Hautzinger M Die Plazebokontrollgruppe in der Psychotherapieforschung Psychotherapie 2001 6 199 204

[B24] Baskin TW Tierney SC Minami T Wampold BE Establishing specificity in psychotherapy: a meta-analysis of structural equivalence of placebo controls J Consult Clin Psychol 2003 71 6 973 9 14622072 10.1037/0022-006X.71.6.973

[B25] Hegerl U Hautzinger M Mergl R Kohnen R Schutze M Scheunemann W Allgaier AK Coyne J Henkel V Effects of pharmacotherapy and psychotherapy in depressed primary-care patients: a randomized, controlled trial including a patients' choice arm Int J of Neuropsychopharm 2010 13 1 31 44 10.1017/S1461145709000224 19341510

[B26] Hautzinger M Welz S Kurz- und längerfristige Wirksamkeit psychologischer Interventionen bei Depressionen im Alter Zeitschr f Klin Psych u Psychoth 2008 37 52 60

[B27] Hoffmann SO Rudolf G Bernhard S Unerwünschte und schädliche Wirkungen von Psychotherapie Psychotherapeut 2008 53 4 16 10.1007/s00278-007-0578-2

[B28] Hoyer J Helbig S Wittchen HU Experiences with Psychotherapy for Depression in Routine Care: a Naturalistic Patient Survey in Germany Clin Psychol Psychother 2006 13 414 421 10.1002/cpp.504

[B29] Markowitz JC Interpersonal psychotherapy for chronic depression J Clin Psychol 2003 59 8 847 858 10.1002/jclp.10177 12858426

[B30] Rush AJ Gullion CM Basco MR Jarrett RB Trivedi MH The Inventory of Depressive Symptomatology (IDS): psychometric properties Psychol Med 1996 26 3 477 486 10.1017/S0033291700035558 8733206

[B31] Rush AJ Trivedi MH Carmody TJ Ibrahim HM Markowitz JC Keitner GI Kornstein SG Arnow B Klein DN Manber R Dunner DL Gelenberg AJ Kocsis JH Nemeroff CB Fawcett J Thase ME Russell JM Jody DN BORIAN FE Keller MB Self-reported depressive symptom measures: sensitivity to detecting change in a randomized, controlled trial of chronically depressed, nonpsychotic outpatients Neuropsychopharmacology 2005 30 2 405 416 10.1038/sj.npp.1300614 15578008

[B32] Derogatis LR Melisaratos N The Brief Symptom Inventory: an introductory report Psychol Med 1983 13 3 595 605 10.1017/S0033291700048017 6622612

[B33] Spitzer RL Kroenke K Williams JB Lowe B A brief measure for assessing generalized anxiety disorder: the GAD-7 Arch Intern Med 2006 166 10 1092 1097 10.1001/archinte.166.10.1092 16717171

[B34] Ware JE Jr Sherbourne CD The MOS 36-item short-form health survey (SF-36). I. Conceptual framework and item selection Med Care 1992 30 6 473 483 10.1097/00005650-199206000-00002 1593914

[B35] Ware J Jr Kosinski M Keller SD A 12-Item Short-Form Health Survey: construction of scales and preliminary tests of reliability and validity Med Care 1996 34 3 220 33 10.1097/00005650-199603000-00003 8628042

[B36] Gandek B Ware JE Aaronson NK Apolone G Bjorner JB Brazier JE Bullinger M Kaasa S Leplege A Prieto L Sullivan M Cross-validation of item selection and scoring for the SF-12 Health Survey in nine countries: results from the IQOLA Project. International Quality of Life Assessment J Clin Epidemiol 1998 51 11 1171 1178 10.1016/S0895-4356(98)00109-7 9817135

[B37] Sugar CA Sturm R Lee TT Sherbourne CD Olshen RA Wells KB Lenert LA Empirically defined health states for depression from the SF-12 Health Serv Res 1998 33 911 28 9776942 PMC1070293

[B38] Jenkinson C Layte R Jenkinson D Lawrence K Petersen S Paice C Stradling J A shorter form health survey: can the SF-12 replicate results from the SF-36 in longitudinal studies? J Public Health Med 1997 19 2 179 86 9243433 10.1093/oxfordjournals.pubmed.a024606

[B39] Hunt SM McKenna SP The QLDS: a scale for the measurement of quality of life in depression Health Policy 1992 22 3 307 319 10.1016/0168-8510(92)90004-U 10122730

[B40] Whalley D McKenna SP Measuring quality of life in patients with depression or anxiety Pharmacoeconomics 1995 8 4 305 315 10.2165/00019053-199508040-00005 10155672

[B41] Horowitz L Strauß B Kordy H Inventar zur Erfassung interpersonaler Probleme 2000 Göttingen: Belz 10593141

[B42] Duschek S Schandry R Hege B SASS, Soziale Aktivität Selbstbeurteilungs-Skala 2003 Göttingen: Belz

[B43] Weissman MM Olfson M Gameroff MJ Feder A Fuentes M A comparison of three scales for assessing social functioning in primary care Am J Psychiatry 2001 158 3 460 466 10.1176/appi.ajp.158.3.460 11229989

[B44] Bernstein DP Fink L Handelsman L Foote J Lovejoy M Wenzel K Sapareto E Ruggiero J Initial reliability and validity of a new retrospective measure of child abuse and neglect Am J Psychiatry 1994 151 8 1132 1136 8037246 10.1176/ajp.151.8.1132

[B45] Bremner JD Vermetten E Mazure CM Development and preliminary psychometric properties of an instrument for the measurement of childhood trauma: the Early Trauma Inventory Depress Anxiety 2000 12 1 1 12 10.1002/1520-6394(2000)12:1<1::AID-DA1>3.0.CO;2-W 10999240

[B46] Bremner JD Bolus R Mayer EA Psychometric properties of the Early Trauma Inventory-Self Report J Nerv Ment Dis 2007 195 3 211 218 10.1097/01.nmd.0000243824.84651.6c 17468680 PMC3229091

[B47] Luborsky L McLellan AT Woody GE O'Brien CP Auerbach A Therapist success and its determinants Arch Gen Psychiatry 1985 42 6 602 611 4004503 10.1001/archpsyc.1985.01790290084010

[B48] Hatcher RL Barends AW Patients' view of the alliance of psychotherapy: exploratory factor analysis of three alliance measures J Consult Clin Psychol 1996 64 6 1326 1336 8991319 10.1037//0022-006x.64.6.1326

[B49] Martin DJ Garske JP Davis MK Relation of the therapeutic alliance with outcome and other variables: a meta-analytic review J Consult Clin Psychol 2000 68 3 438 450 10883561

[B50] Luborsky L A pattern-setting therapeutic alliance study revisited Psychotherapy Research 2000 10 1 17 28 10.1080/713663591

[B51] First M Spitzer RL Gibbon M Williams JB Structured Clinical Interview for DSM-IV Axis I Disorders 1997 New York: American Psychiatric Publ

[B52] First M Gibbon M Spitzer RL Williams J Benjamin L Structured Clinical Interview for DSM-IV Axis II Personality Disorders (SCID-II) 1997 New York: American Psychiatric Press

[B53] Thase ME Commentary. What is the investigator allegiance effect and what should we do about it? Clin Psych: Scien a Pract 1999 6 1 113 115 10.1093/clipsy/6.1.113

[B54] National Institute for Clinical Excellence Depression: management of depression in primary and secondary care Clinical Guideline 23 2004 London: NICE

